# The developmental trajectories of executive function from adolescence to old age

**DOI:** 10.1038/s41598-020-80866-1

**Published:** 2021-01-14

**Authors:** Heather J. Ferguson, Victoria E. A. Brunsdon, Elisabeth E. F. Bradford

**Affiliations:** 1 School of Psychology, University of Kent, Canterbury, UK; 2 School of Psychology, University of Dundee, Dundee, UK; 3 Present Address: School of Psychology, University of Kent, Canterbury, CT2 7NP UK

**Keywords:** Cognitive ageing, Learning and memory

## Abstract

Executive functions demonstrate variable developmental and aging profiles, with protracted development into early adulthood and declines in older age. However, relatively few studies have specifically included middle-aged adults in investigations of age-related differences in executive functions. This study explored the age-related differences in executive function from late childhood through to old age, allowing a more informed understanding of executive functions across the lifespan. Three hundred and fifty participants aged 10 to 86 years-old completed a battery of tasks assessing the specific roles of inhibitory control, working memory, cognitive flexibility, and planning. Results highlighted continued improvement in working memory capacity across adolescence and into young adulthood, followed by declines in both working memory and inhibitory control, beginning from as early as 30–40 years old and continuing into older age. Analyses of planning abilities showed continued improvement across adolescence and into young adulthood, followed by a decline in abilities across adulthood, with a small (positive) change in older age. Interestingly, a dissociation was found for cognitive flexibility; switch costs decreased, yet mixing costs increased across the lifespan. The results provide a description of the developmental differences in inhibitory control, working memory, cognitive flexibility and planning, above any effects of IQ or SES, and highlight the importance of including middle-aged adults in studies seeking to establish a more comprehensive picture of age-related differences in executive function.

## Introduction

Executive functions (EF) are high-level cognitive processes that include planning, initiation, shifting, monitoring, and inhibition of behaviours^1^. EFs play an important role in our everyday life, allowing us to focus attention on specific tasks, to engage in successful problem solving, and to plan for the future. EFs demonstrate variable developmental and aging profiles (e.g.,^2,3^), with protracted development into early adulthood and a decline into older age that is associated with structural and functional changes in the prefrontal cortex^4–10^. The majority of these studies have compared dichotomous young/old adult age groups, and few studies include middle-aged adults or adolescents in investigations of age-related changes in EF (c.f.^11–13^ who included middle-aged adults). Therefore, many open questions remain about how development changes across the lifespan, and whether these effects are consistent across multiple components of EF. We address this by exploring how different components of EF develop and change across the lifespan, from late childhood through to old age. Specifically, we tested whether four key components of EF (inhibition, working memory, cognitive flexibility and planning) show parallel or distinct developmental trajectories, and aimed to describe any age-related changes in multiple EFs.

EFs begin to emerge early in infancy, with basic skills needed for EFs emerging before three years of age, and more specific skills developing into early childhood^14^. It has been suggested that each component of EF develops at its own rate across childhood and adolescence, reaching maturity at different ages (see^1^). For instance, cognitive flexibility has been shown to emerge between the ages of 3 and 4 years old, becoming more complex between the ages of 7 and 9 years old, and reaching adult-like levels by age ~ 12^15–17^; in contrast, Zelazo et al.^18^ found that cognitive flexibility abilities continue to improve between the ages of 20 and 29 years old, suggesting prolonged development of these abilities into young adulthood, and highlighting the importance of using different approaches and tasks to assess EF abilities, providing further insight into when these abilities reach maturity. Working memory, inhibition, and planning have been shown to continue to develop throughout childhood and adolescence, and in some circumstances (e.g., task dependent), have also been shown to continue to develop into young adulthood (e.g.,^19–23^). The protracted development of EFs across childhood and adolescence is associated with neurological changes, particularly the development of the prefrontal cortex (e.g.,^4,24,25^). Given this, adolescence is a critical period to study, allowing further examination of the continued development of EFs beyond childhood and into early adulthood to establish when these components of EF reach maturity.

Cognitive performance peaks in young adulthood (e.g.,^26^), with declines emerging as early as 20 or 30 years old, including declines across adulthood in speed of processing^27–30^, reasoning^29,30^, face processing^31^, fluid intelligence^26,27^, crystallized intelligence^26,27^, working memory^26,28,32,33^, verbal and visuospatial memory^34^, and long-term memory^27,28^. There is a vast amount of heterogeneity in regards to when cognitive abilities peak and decline. For example, aspects of short-term memory decline from 18 years of age, working memory declines in the 30 s, and vocabulary peaks in the 40 s or even later^26^. In contrast, other aspects of cognition, such as autobiographical memory and semantic knowledge, remain relatively stable across adulthood^35,36^.

These findings raise the question of whether different components of EFs, specifically inhibition, working memory, cognitive flexibility, and planning, are stable across adulthood and decline in older age, or whether age-related declines in EFs begin soon after maturity in early adulthood. Studies have largely established that working memory reaches a peak at 30 years old and declines thereafter^26,32,33,37^. In addition, inhibitory control is poorest in younger children, improves in adulthood, and declines in older age^38^; however middle-aged adults were omitted from this study, so it is not clear when these declines started to emerge. Overall, there is a paucity of research specifically focussing on multiple components of EF across the lifespan, with studies into aging often limited in their focus due to comparing dichotomous ‘young’ versus ‘old’ adult groups (i.e. few studies include adolescents or middle-age adults in their lifespan sample). This approach means that important evidence is scarce to draw conclusions on the extended developmental trajectory of EF or earlier signs of decline. A notable exception to this is the Cognitive Battery of assessments developed as part of the National Institutes Health Toolbox in the U.S.A (NIHTB-CB;^18,39,40^). The NIHTB-CB sought to establish a series of tasks that could be used to assess cognitive function abilities across different populations of individuals, suitable for use in individuals aged from three to 85 years old, and includes measures of inhibitory control, cognitive flexibility, and working memory. Results from the NIHTB-CB support suggestions of an inverted-U-shaped curve in development of a number of EF abilities, including inhibition, cognitive flexibility, and working memory, with abilities first rising across childhood, and falling in later adulthood^18,41,42^. Ferriera et al.^43^ also investigated EF abilities in a specific cohort of healthy middle-aged adults, with results highlighting very early declines in EF before the age of 50; other studies that have included middle-aged adults in a broader adult sample have reported a linear decline across adulthood which is steeper among participants aged 65 + (e.g.,^13^).

Further to these behavioural studies, neuroimaging has revealed changes in both the structure and function of brain regions that underlie EFs in middle-age and older adulthood^44,45^, which is highly likely to impact EF performance in these age ranges. The studies cited above have provided important insights into the developmental trajectories of EF capacities across the lifespan, including highlighting the limited studies that have included middle-aged adults in investigations of EFs and, importantly, included analysing age as a continuous measure to track development throughout adulthood (c.f.^11–13,46^). More often, even when studies have included middle-age adults, they have analysed effects of age between groups rather than as a continuous predictor (e.g.,^47,48^), or rely on correlation or regression analyses to model only linear trends (e.g.,^46^). As illustrated, studies with middle-aged adults are essential to gain a comprehensive picture of the development of EFs throughout adulthood, to allow pinpointing of when declines in EFs first emerge, and whether the patterns of decline in early adulthood, as shown in other cognitive abilities, are also evident across the different components of EF across different paradigms, or whether they are limited to specific components. Conducting studies with a continuous age sample also provides vital insights to inform theories of healthy and abnormal aging, as, for example, the first pathophysiological changes can commence up to 20 years before a diagnosis of dementia^49^.

Older age is associated with significant declines in EF, including working memory (e.g.,^50^), inhibition (e.g.,^51^), planning (e.g.,^52^), and cognitive flexibility (e.g.,^53^). Additionally, different aspects of cognitive flexibility show distinct age-related effects. Mixing costs are greater in older adults (e.g.,^54–58^); however, there are mixed results in regard to switch costs, with some studies reporting an age-related increase (e.g.,^59^), a U-shaped trajectory^53^, or no age-related differences (e.g.,^58, 59^), most likely due to differences in paradigms. Age-related effects in EFs are thought to be relatively robust, and have been associated with changes in the frontal lobes, specifically age-related volume reduction in the prefrontal cortex^60^. There are some conflicting findings in the literature regarding age-related declines in EF, perhaps because many studies do not account for general slowing in response latencies (see^61^, for a discussion). When accounting for this general slowing, Verhaeghen^61^ failed to find evidence for specific age-related declines in inhibition and local task-shifting costs (termed switch costs herein), but found evidence for age-related declines in global task-shifting costs (termed mixing costs herein). Verhaeghen suggested that mixing costs reflect a dual-task cost, with dual-tasks affecting older adults more^62^. Thus, it is important for studies examining effects of cognitive decline in older age to account for age-related changes in response speed, to be sure that effects reflect true changes in executive capacities rather than more general slowing in response latencies.

In addition to age, several factors have been linked to cognitive decline, including genetics, health status, physical activity, socio-economic status (SES), IQ, and physical fitness (e.g.,^63–68^). Childhood SES has been consistently associated with EF^56,69–71^, with lower SES predicting poorer performance on tasks of EF in childhood^72^. Less is known about the link between adult SES and EF^73^. IQ is another factor that has been associated with EF, particularly with working memory^74^. IQ and EFs are dissociable yet related in childhood^75^, with evidence that inhibitory control and cognitive flexibility are related to IQ during childhood^76^. In adolescence, working memory is highly correlated with IQ, but inhibition and cognitive flexibility are not^54^. In older adults, IQ has been shown to be related to working memory, verbal fluency, inhibition, and cognitive flexibility^77^. Given that IQ and SES are related to EF abilities, the current study controlled for these factors in analysis, allowing us to assess the role of age in predicting differences in EFs, beyond effects of IQ and SES.

EFs play a critical role in everyday life, allowing individuals to plan ahead, focus their attention, and switch between different tasks. They play a key role in allowing individuals to maintain effective levels of independent functioning, and better EF abilities have been associated with improved self-reported quality of life in older age^1,78^. Further, deficits in EF abilities have been associated with issues with obesity^79^, social problems^80,81^, and lower levels of productivity^82^. It is therefore important to further our understanding of how these EF abilities continue to change and differ across the lifespan—contributing to our understanding of age-related cognitive changes—which ultimately may be able to provide insight into the optimum age at which cognitive training interventions could be utilized to help maintain real-world functioning across individuals.

The current study investigated how multiple components of EF differ across the lifespan, in a large, community-based sample of 350 10- to 86-year-olds, allowing differences across adolescence, early adulthood, middle adulthood, and older adulthood to be examined within one study. The study focussed on four components of EF: planning, inhibition (also termed inhibitory control and response inhibition), working memory, and cognitive flexibility (also called set shifting or mental flexibility). It is largely accepted that inhibitory control, working memory, and cognitive flexibility form the core components of EF abilities, reflecting largely (but not entirely) separable processes^83^. In the current study we also included a more complex aspect of EF, planning abilities. The ability to plan is a complex executive skill^94,102^ that plays an important role in daily living, such as the ability to identify a goal and subsequently planning and executing the steps needed to attain that goal^72,83^. It is noted that planning abilities themselves, whilst considered an aspect of EF, may require activation of other EFs, including inhibitory control and working memory in order to produce successful outcomes^72,83^. Given this, the inclusion of a measure of planning abilities in the current study allowed further insight into how planning capacities may change across the lifespan, and whether we are able to establish a relationship between ‘core’ EF abilities and planning capacities within this lifespan sample.

The aim of this study was to explore the developmental trajectories of these four components of EF, to identify when age-related differences emerge. A cross-sectional design was utilized, to provide insight into differences that can be established across different age cohorts in task performance; importantly, to address our research question, we selected tasks that were appropriate for all participants from 10 to 86 years of age, allowing direct comparisons in task performance to be made across different ages. We used curvilinear regression modelling to establish the shape and trajectory of change across ages for each EF. Due to research suggesting that some components of EF may be related to IQ and SES, we also controlled for the effects of IQ and socio-economic status.

We predicted, firstly, that these components of EF would continue to develop throughout adolescence, indicated by an improvement in performance across tasks up to ~ 30 years of age. Second, we predicted that there would be age-related declines in EF from ~ 50 years of age onwards^43^. Third, we explored whether this decline in EFs would start earlier in adulthood (i.e. between 30 and 50 years of age). We did not stipulate specific predictions in this middle age range due to the dearth of research in adulthood. Instead, we modelled and tested the fit of linear, quadratic and cubic age relationships for each component of EF. Note that each statistical model can represent multiple patterns/directions of effects, however we define our predictions for the linear, quadratic and cubic fit models used here based on existing research on cognitive development and decline with age. We posited that a predicted linear age relationship would indicate either an improvement or decline in EF from adolescence to older age. We predicted that a quadratic age relationship would indicate a developmental improvement in EF in adolescence through to young adulthood, and a decline in EF throughout adulthood. A predicted cubic age relationship would indicate a developmental improvement in EF in adolescence through to young adulthood, a decline in EF across adulthood, and a further steeper decline in EF in older age. Finally, in line with previous research (e.g.,^84^) we predicted that the different aspects of cognitive flexibility would should show distinct effects: we predicted that switching costs (i.e., changing task sets) would not show any age-related changes, but mixing costs (i.e., maintaining multiple task sets) would show an increase across adulthood (e.g.,^84, 85^).

## Materials and method

### Participants

The sample consisted of 354 participants who were recruited from the community, via newspaper/radio adverts, social media, and an institutional research participation database, as part of the CogSoCoAGE project. Two participants were excluded due to low IQ (< 70), one participant was excluded due to being a non-native English speaker, and one participant’s data was lost due to computer failure. The final sample consisted of 350 participants (10–86 years-old; 232 females, 118 males). Table 1 provides a summary of the sample and Table 2 details the demographic characteristics of the CogSoCoAGE sample, each divided into five age groups for illustrative purposes. All participants were native English-speakers, had normal or corrected-to-normal vision, had no known neurological disorders, and had no mental health or autism spectrum disorder diagnoses. The Ethical Committee of the School of Psychology, University of Kent, approved the study, and all methods were carried out in accordance with EU guidelines and regulations. Informed consent was obtained from all participants; for participants under 18 years of age, consent was additionally sought from a parent or legal guardian.

**Table 1:** Summary of the CogSoCoAGE sample.

	All (10–86 years old)	Adolescents (10–17 years old)	Young adults (18–29 years old)	Adults (30–49 years old)	Middle-aged adults (50–64 years old)	Older adults (65–86 years old)
N	350	62	60	76	74	78
**Age (years)**						
Mean (SD)	43.22 (22.14)	13.34 (2.39)	22.63 (3.59)	40.00 (5.62)	57.19 (4.28)	72.71 (5.64)
**Gender**						
F:M ratio	232:118	29:33	39:21	62:14	53:21	49:29
**IQ**						
Verbal IQ	109.65 (12.38)	105.74 (10.07)	108.13 (11.06)	103.05 (9.82)	109.86 (11.50)	120.14 (11.52)
Performance IQ	108.48 (12.92)	107.27 (13.47)	104.70 (12.18)	105.45 (11.57)	110.18 (13.12)	120.66 (11.30)
Full scale IQ	110.35 (12.19)	107.35 (11.07)	107.45 (10.75)	104.70 (9.81)	110.18 (11.83)	120.65 (10.43)
**SES index**						
Mean (SD)	13.74 (3.70)	14.79 (3.41)	11.18 (3.82)	14.56 (3.78)	13.91 (3.35)	13.91 (3.22)

**Table 2:** Demographic characteristics of the CogSoCoAGE sample.

Characteristic (N (%))	All	Adolescents	Young adults	Adults	Middle-aged adults	Older adults
**Ethnicity**						
White	316 (90.3)	52 (83.9)	46 (76.6)	71 (93.4)	70 (94.6)	77 (98.7)
Mixed/multiple ethnic groups	13 (3.7)	7 (11.3)	3 (5.0)	1 (1.3)	2 (2.7)	0
Asian/British Asian	8 (2.3)	0	5 (8.3)	3 (4.0)	0	0
Black/African/Caribbean/Black British	3 (0.9)	0	2 (3.3)	1 (1.3)	0	0
Other ethnic group	3 (0.9)	0	1 (1.7)	0	1 (1.4)	1 (1.3)
Not stated	7 (2.0)	3 (4.8)	3 (5.0)	0	1 (1.4)	0
**Education**						
GCSEs	35 (10.0)	7 (11.3)	1 (1.7)	9 (11.8)	10 (13.5)	8 (10.3)
A-Levels	60 (17.1)	10 (16.1)	32 (53.3)	6 (7.9)	8 (10.8)	4 (5.1)
Undergraduate degree	91 (26.0)	14 (22.6)	19 (31.7)	18 (23.7)	18 (24.3)	22 (28.2)
Postgraduate degree	90 (25.7)	16 (25.8)	5 (8.3)	25 (32.9)	21 (28.4)	23 (29.5)
Other	64 (18.3)	12 (19.4)	1 (1.7)	17 (22.4)	15 (20.3)	19 (24.4)
No qualifications	2 (0.6)	0	0	1 (1.3)	0	1 (1.3)
Not stated	8 (2.3)	3 (4.8)	2 (3.3)	0	0	1 (1.3)
**Household income**						
< £9999	28 (8.0)	3 (4.84)	10 (16.67)	3 (3.95)	5 (6.76)	7 (8.97)
£10,000–£19,999	44 (12.3)	2 (3.23)	7 (11.67)	9 (11.84)	8 (10.81)	17 (21.79)
£20,000–£29,000	49 (14.0)	6 (9.68)	7 (11.67)	6 (7.89)	15 (20.27)	15 (19.23)
£30,000–£39,000	52 (14.8)	2 (3.23)	7 (11.67)	12 (15.79)	15 (20.27)	16 (20.51)
£40,000–£49,000	56 (16.0)	15 (24.19)	7 (11.67)	11 (14.47)	10 (13.51)	13 (16.67)
£50,000–£69,999	56 (16.0)	16 (25.81)	7 (11.67)	18 (23.68)	11 (14.86)	4 (5.13)
£70,000+	49 (14.0)	13 (20.97)	9 (15.00)	16 (21.05)	7 (9.46)	4 (5.13)
Not stated	17 (4.9)	5 (8.06)	6 (10.00)	1 (1.32)	3 (4.05)	2 (2.56)
**Occupational class**						
Higher managerial, administrative and professional	55 (15.7)	9 (14.52)	1 (1.67)	11 (14.47)	13 (17.57)	21 (26.92)
Lower managerial, administrative and professional	124 (35.4)	21 (33.87)	8 (13.33)	31 (40.79)	30 (40.54)	34 (43.59)
Intermediate occupations	76 (21.7)	21 (33.87)	5 (8.33)	19 (25.00)	16 (21.62)	15 (19.23)
Small employers and own account workers	9 (2.6)	0	2 (3.33)	2 (2.63)	2 (2.70)	3 (3.85)
Lower supervisory and technical occupations	4 (1.2)	1 (1.61)	1 (1.67)	1 (1.32)	1 (1.35)	0
Semi-routine occupations	38 (10.9)	7 (11.29)	7 (11.67)	10 (13.16)	9 (12.16)	5 (6.41)
Routine occupations	12 (3.4)	0	11 (18.33)	1 (1.32)	0	0
Never worked and long-term unemployed	0	0	0	0	0	0
Full-time student	21 (6.0)	0	19 (31.67)	1 (1.32)	1 (1.35)	0
Not stated	11 (3.1)	3 (4.84)	6 (10.00)	0	2 (2.70)	0

### Measures

#### Socio-economic status

Participants (if aged over 18) and parents of participants (if aged under 18) reported on their level of education, the household income, and their occupation (job title and industry). Occupational class was coded using the derivation tables provided by the Office for National Statistics^116^ using the simplified National Statistics Socio-Economic Classification (NS-SEC) based on Standard Occupational Classification 2010 (SOC2010). To calculate an SES index, education level was coded on a scale 1–6, and household income and occupational class were coded on a scale 1–7. These three scores were summed to derive an SES index between 3 and 20^86^, with lower scores indicating lower SES. In our sample, scores ranged from 5 to 20.

#### IQ

Intellectual ability was assessed using the Wechsler Abbreviated Scale of Intelligence-Second Edition (WASI;^87^). The WASI-II comprises of four subtests as a measure of intelligence for individuals aged 6–90 years old. The Vocabulary and Similarities subtests estimated a verbal IQ score. The Block Design and Matrix Reasoning subtests estimated a performance IQ score. Full-scale IQ comprised of both verbal and performance IQ.

#### Stroop colour-word task

**Figure 1:**
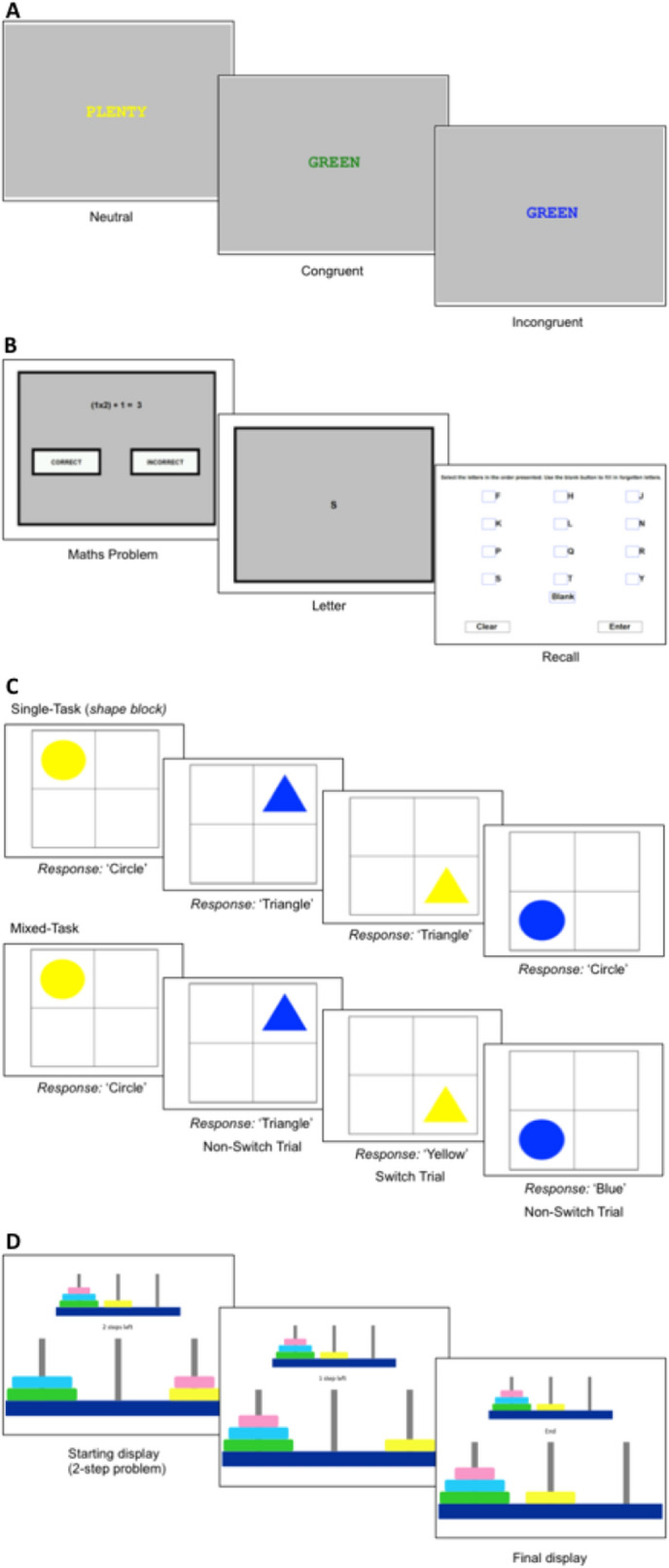
Illustrations of the stimuli and procedure employed in each of the four EF tasks: (**A**) Stroop colour-word task; (**B**) operation span; (**C**) task switching; (**D**) Tower of Hanoi.

A modified version of a standard Stroop Colour-Word task^88^ was used as a measure of inhibition. The words were printed in red, green, blue, or yellow for all trials and were printed on a grey background. The words used in both congruent and incongruent trials were “RED”, “GREEN”, “BLUE”, and “YELLOW”. For congruent trials, the colour word matched the printed colour (i.e., “RED” printed in red). For incongruent trials, the colour word did not match the printed colour (i.e., “RED” printed in green). For filler trials, the non-colour words were matched for length and frequency to the colour words. The filler words used were “TAX”, “CHIEF”, “MEET”, and “PLENTY”. The word stimuli were presented in the middle of the screen in font type Courier New and font size 28. See Fig. 1 for example stimuli.

Participants first completed 20 practice trials, which consisted of ten filler and ten congruent trials in a pseudo-randomised order. Participants were told that they would see a word and they were instructed to identify the colour of the word as fast as possible using a button-box (i.e., RED printed in green; participants press ‘green’ button). The experimental trials consisted of 50 congruent trials, 50 incongruent trials, and 50 filler trials presented in a pseudo-randomised order, in which the same colour word, the same printed colour, or the same colour word/printed colour could not appear on two consecutive trials to avoid priming effects. A blank screen appeared for 1000 ms at the start of the experimental trials. After the participant made a response, the next trial was started immediately.

Response times for filler, congruent and incongruent trials were calculated for accurate responses that were made 200 ms after stimuli onset and were within 2.5 SDs of each participant’s overall trial mean. The dependent variable was the Stroop congruency effect (incongruent trial mean RT minus congruent trial mean RT). In addition, we accounted for age-related slowing and declines in information processing speed, which led to positive skew and high kurtosis in reaction times, by log-transforming reaction times for each trial before calculating the Stroop congruency effect. The log-transformation of the Stroop congruency effect reduced skew and kurtosis (untransformed skew = 1.84, kurtosis = 8.84; log-transformed skew = 0.69, kurtosis = 3.44). The log-transformed Stroop congruency effects were reverse scored so that a higher score indicated better performance to aid interpretation of results alongside other measures. Internal consistency was excellent (Cronbach’s alpha = 0.99) and the average inter-item correlation was ideal (r = 0.53).

#### Operation span

This task was adapted from Unsworth et al.’s^89^ automated operation span task (OSpan) as a measure of working memory, which was based on the original OSpan task by Turner and Engle^90^. Participants were required to solve maths equations while remembering a sequence of letters. The letters used were F, H, J, K, L, N, P, Q, R, S, T, and Y. See Fig. 1 for example stimuli.

There were three practice blocks. The first practice block was a simple letter span. A single letter appeared in the middle of the screen for 800 ms. A two-letter span was used for two trials, and a three-letter span was used for a further two trials. At recall, participants were required to recall the letter sequence in the correct order by clicking a box next to the appropriate letter presented in a 4 × 3 matrix. After clicking a box, a number appeared that represented the position of the letter in the sequence. A ‘blank’ box was also presented and participants were told to click this box if they could not remember the letter in the sequence. Participants could also click a ‘clear’ box to clear responses. The letters clicked also appeared at the bottom of the screen. To finish the letter recall stage, participants clicked a box labelled ‘enter’. This recall phase was untimed. After the recall phase, participants were given feedback about how many letters they recalled correctly.

The second practice block introduced the maths equations. A maths equation was presented on screen (e.g., (2 × 1) + 1 = 3) along with a ‘correct’ box and an ‘incorrect’ box. Participants were required to identify whether the maths equation was correct or incorrect by clicking the appropriate box. Accuracy feedback was given. There were three trials in this second practice block.

In the last practice block, participants completed both the maths section and letter recall section together. The maths equation was presented first, and once participants had responded to the problem, a letter to be recalled appeared in the middle of the screen for 800 ms. This equation-letter sequence was repeated twice to create a two-letter span in this final practice block. The letter recall screen with the 4 × 3 letter matrix was then presented. Participants completed three full practice trials, and were given feedback on how many letters they recalled correctly and how many errors they made on the maths problems.

The experimental trials consisted of three trials for each of 2 to 7 letter spans (randomised). This made a total of 18 trials with 81 maths problems and 81 letters. Participants were encouraged to keep their maths accuracy at or above 85% at all times. During recall, a percentage in red was presented in the upper right-hand corner of the screen, indicating the percentage accuracy for the maths problems.

An absolute OSpan score was calculated as the sum of all perfectly recalled sets. A partial OSpan score was also calculated as the total number of letters recalled in the correct position. The absolute and partial OSpan scores were highly correlated (r = 0.92, *p* < 0.001) and due to the recommendations of Unsworth et al.^89^, the partial OSpan score was used as the dependent variable. Internal consistency was good (Cronbach’s alpha = 0.85) and the average inter-item correlation was ideal (r = 0.25).

#### Task switching

The task was adapted^91,92^ as a measure of cognitive flexibility. Participants were presented with a 2 × 2 matrix on a computer screen. Stimuli were presented one-by-one in the four quadrants of the screen, beginning in the upper-left quadrant and rotating in a clockwise manner. The stimuli were coloured-shapes (circle/triangle, in blue/yellow) that appeared in the quadrant. See Fig. 1 for example stimuli. The same shape/colour combination did not appear on consecutive trials (i.e., a blue triangle could not appear in consecutive trials). Participants’ task was to decide whether the shape was a circle or a triangle, and whether the colour was blue or yellow, dependent on trial-type (see descriptions below). Participants used a button box to respond, pressing the left-hand button for circle/blue and the right-hand button for triangle/yellow. Participants were instructed to respond as fast and as accurately as possible. The next stimulus was presented 150 ms after a key press or after a timeout of 5000 ms. Participants received feedback about their accuracy after practice trials and repeated the practice block if their accuracy was less than 80%.

In the single-task, there were 16 practice trials and 32 experimental trials per block. Participants had to identify whether the shape was a circle or a triangle in one block, and whether the colour was blue or yellow in a second block (single-task trials).

In the mixed-task, there were 16 practice trials, and four blocks of 32 experimental trials. Participants had to indicate whether the shape was a circle or a triangle when the coloured-shape appeared in the top two quadrants, and whether the colour was blue or yellow if the coloured-shape appeared in the bottom two quadrants. Categorising the coloured-shape in the upper left to upper right quadrant, or in the lower right to lower left quadrant did not require switching to a new category (i.e., non-switch trials). However, categorising the coloured-shape in the upper right to lower right quadrant, or in the lower left to upper left quadrant required switching to a new category (from shape to colours, and vice versa, i.e., switch trials). Switch and non-switch trials alternated predictably within these blocks.

Response times were calculated for accurate responses that were made 200 ms after stimuli onset, and were within 2.5 SDs of each participant’s overall trial mean. A switch cost of task-set switching was calculated by subtracting the mean response time for non-switch trials from the mean response time for switch trials in the mixed-task. A mixing cost (indicating maintenance of two task-sets) was calculated by subtracting the mean single-task trial response time from the mean non-switch response time in the mixed-task. To account for age-related slowing and declines in information processing speed, trial level response times were log-transformed before calculating a switch cost and mixing cost. The log-transformation reduced skew and kurtosis for switch cost (untransformed skew = 0.47, kurtosis = 3.25; log-transformed skew = 0.17, kurtosis = 2.54) and mixing cost (untransformed skew = 0.91, kurtosis = 3.39; log-transformed skew = 0.41, kurtosis = 2.83). The log-transformed switch and mixing costs were reverse scored so that a higher score indicated better performance. Internal consistency was excellent for both the single and mixed-task (both Cronbach’s alpha = 0.98). The average inter-item correlation for the single-task (r = 0.49) and for the mixed-task (r = 0.34) was ideal.

#### Tower of Hanoi

The Tower of Hanoi was used as a measure of planning (based on script obtained from: https://step.talkbank.org/scripts-plus/TOHx.zip). The Tower of Hanoi required the mouse-controlled movement of different-sized disks across three pegs from an initial state to a target state in a pre-defined number of steps. Participants were presented with three pegs (left, centre, right) and four disks; pink, yellow, blue and green, in increasing size. The target state was shown on the top-centre of the screen and was smaller than the initial state configuration. The initial state was presented on the bottom-centre of the screen. The number of steps remaining was shown in the centre of the screen. Participants were told that they needed to move the disks from their current positions on the bottom of the screen to match the target state in the given number of steps without placing larger disks on top of smaller disks. See Fig. 1 for example stimuli.

Participants first completed three practice trials: one one-step and two two-step problems. Participants continued to 16 experimental trials, which took three- to ten-steps to complete, with two trials at each step. Before the start of each trial, participants were told how many steps were required to complete each trial. During the trials, participants clicked on the disk that they wanted to move and this disk then turned red. The participant then clicked on the rod that they wanted to move the disk to. If the incorrect rod was selected, then an error message was shown and the participant restarted that trial. If the participant made five incorrect movements in a row then the task automatically ended. If the correct disk and rod were selected, then the selected disk moved to the selected rod and the participant moved on to the next step.

The dependent variable was an overall Tower of Hanoi score that used the traditional absolute scoring method, and was the sum of all perfectly completed trials (i.e., score of 5 for a trial with 5 steps completed perfectly with no errors). Internal consistency was acceptable (Cronbach’s alpha = 0.80) and the average inter-item correlation was ideal (r = 0.20).

### Procedure

Participants attended one or two visits to the university to complete the 5 h testing session, which included questionnaires on behaviour and demographic information, computer-based testing to assess cognitive and social skills, and an IQ assessment. The order of tasks was counterbalanced over 12 different lists to ensure that order effects were minimised. All tasks reported here were programmed using E-Prime software.

## Results

Analyses were conducted in R version 3.6.0. The datasets and code are available on the Open Science Framework (https://osf.io/qzrwu). Descriptive data on the EF measures are summarised in Table 3, alongside the total number of participants retained per task. For the Stroop task, two participants did not complete the task due to equipment failure and one participant was colour-blind. For the Operation Span, one participant did not complete the task due to equipment failure, 3 participants did not return for their second testing session to complete the task, and 12 participants declined to complete the task or withdrew. For Task Switching, two participants did not complete the task due to equipment failure, 3 participants did not return for their second testing session to complete the task, 10 participants declined to complete the task or withdrew, and two participants’ data was lost due to computer error. For the Tower of Hanoi, two participants did not return for their second testing session to complete the task.

**Table 3:** Descriptive statistics for the executive function measures, showing means and standard deviations in parentheses, divided into five age groups for illustrative purposes.

Executive function measure	N	All	Adolescents	Young adults	Adults	Middle-aged adults	Older adults
**Stroop task (inhibitory control)**^a^							
Filler words RT (log ms)	347	6.89 (.21)	6.88 (.18)	6.72 (.13)	6.81 (.17)	6.93 (.15)	7.07 (.21)
Congruent words RT (log ms)	347	6.86 (.22)	6.84 (.18)	6.67 (.15)	6.79 (.18)	6.92 (.16)	7.05 (.21)
Incongruent words RT (log ms)	347	7.01 (.25)	6.98 (.21)	6.81 (.17)	6.91 (.21)	7.06 (.17)	7.24 (.23)
Congruency effect (log ms)	347	− .14 (.09)	− .14 (.09)	− .14 (.08)	− .13 (.07)	− .14 (.08)	− .19 (.11)
**Operation span (working memory)**							
Absolute score	334	44.60 (18.82)	47.39 (18.11)	52.67 (17.67)	46.85 (16.84)	42.77 (18.43)	35.68 (18.97)
Partial score	334	61.77 (13.82)	64.52 (11.02)	67.51 (9.89)	64.38 (11.37)	59.84 (14.04)	54.47 (16.48)
**Task switching (cognitive flexibility)**^a^							
Single-task trials (log ms)	333	6.42 (.21)	6.38 (.20)	6.27 (.18)	6.36 (.18)	6.49 (.17)	6.59 (.18)
Non-switch trials (log ms)	333	6.88 (.30)	6.77 (.25)	6.64 (.22)	6.84 (.30)	6.99 (.27)	7.10 (.23)
Switch trials (log ms)	333	7.21 (.25)	7.16 (.22)	7.02 (.20)	7.18 (.25)	7.26 (.24)	7.40 (.18)
Switch cost (log ms)	333	− .35 (.16)	− .38 (.16)	− .38 (.18)	− .34 (.15)	− .27 (.16)	− .30 (.17)
Mixing cost (log ms)	333	− .41 (.22)	− .39 (.20)	− .37 (.21)	− .48 (.24)	− .50 (.26)	− .51 (.22)
**Tower of Hanoi (planning)**							
Absolute score	348	49.97 (25.82)	40.16 (20.06)	56.74 (26.53)	55.16 (25.76)	47.90 (25.56)	49.29 (27.37)

^a^Log transformed response times.

### Age-related effects on executive function

A series of regression models were conducted to investigate the relationship between the measures of EF and age, over and above any potential effects of IQ and SES. The models specified the outcome variable as the dependent measure for the specific EF measure, the first predictor variable was age using linear, quadratic or cubic orthogonal polynomial coefficients, and IQ and SES index were included as the second and third predictor variables. Note that quadratic models included both linear and quadratic age coefficients, and cubic models included linear, quadratic and cubic age coefficients.

**Figure 2:**
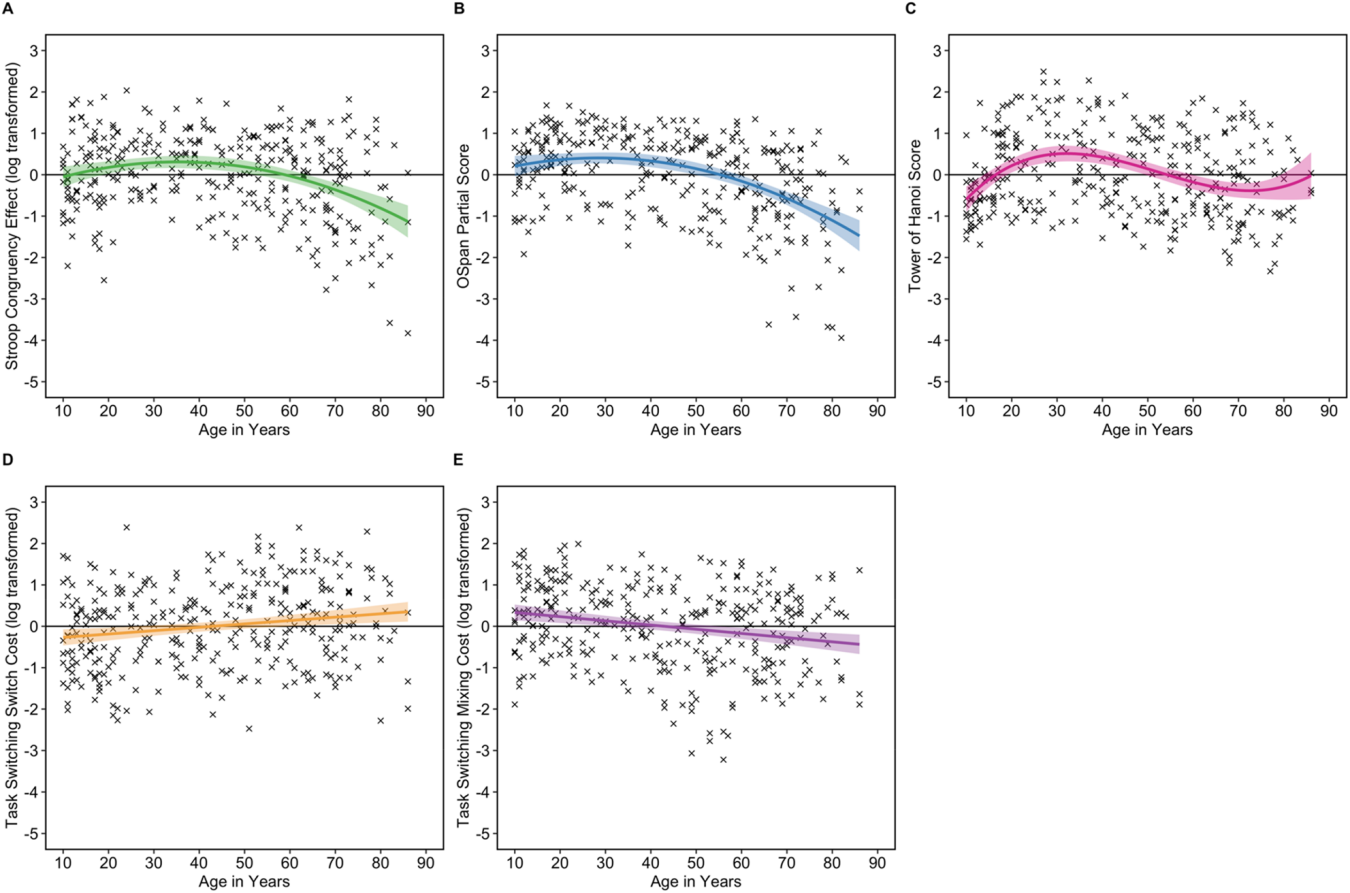
Relationship between age and executive function measures, adjusted for IQ and SES index. (**A**) log-transformed Stroop congruency effect, (**B**) OSpan partial score, (**C**) Tower of Hanoi score, (**D**) log-transformed Task Switching switch cost; and (**E**) log-transformed Task Switching mixing cost. The bold line indicates the best-fitting regression line and the dashed line indicates the 95% confidence intervals (CIs). Stroop congruency effect and Task Switching switch and mixing costs are reversed scored so that a higher value indicates better performance and all variables are z-scored for ease of comparison. *Note*: All measures were adjusted for IQ and SES to be comparable to the described regression models. To adjust for IQ and SES, the residuals were obtained from the regression line fit when fitting each executive function measure as a dependent variable in a linear model and IQ and SES index as predictor variables.

The best fitting model for each EF measure was deduced by comparing several goodness-of-fit indices shown in Table 4. Established goodness-of-fit measures were used to evaluate model fit. The ANOVA test and likelihood test contrasted the simpler model against the more complex model (e.g., the model with linear vs. quadratic age coefficients). If the *p* value was greater than 0.05, then the simpler model was selected as the best fitting model. If the *p* value was less than 0.05, then the more complex model was selected as the best fitting model. Model comparison also used Akaike’s Information Criterion (AIC) and Bayesian Information Criteria (BIC), with increasingly negative values corresponding to increasingly better fitting models. Model selection evaluated these goodness-of-fit indices and the model (linear, quadratic, or cubic model) with the greatest number of goodness-of-fit indices was selected as the overall best fitting model (see Table 4). The model predictions for the overall best fitting models for each EF are plotted in Fig. 2 with the observed data. Analyses for the untransformed variables are reported in Supplementary Materials (S1, S2).

**Table 4:** Goodness-of-fit indices for the executive function models with age as the predictor variable (with linear, quadratic, or cubic age coefficients) for each outcome variable.

Model	Model fit indices							
	ANOVA			Likelihood test			AIC	BIC
	RSS	*F*Δ	*p*	− 2LL	χ^2^	*p*		
**Stroop task (congruency effect)**								
Linear	318.98	–	–	− 471.59	–	–	953.18	972.32
Quadratic^a^	**294.93**	**27.31**	** < 0.001**	− **458.26**	**26.65**	**< 0.001**	**928.53**	**951.50**
Cubic	294.93	0.00	0.990	− 458.26	0.00	0.990	930.53	957.33
**Operation span (partial score)**								
Linear	251.50	–	–	− 421.07	–	–	852.14	871.09
Quadratic^a^	**225.71**	**36.79**	**< 0.001**	− **403.38**	**35.38**	**< 0.001**	818.76	**841.50**
Cubic	224.20	2.15	0.143	− 402.29	2.19	0.139	**818.57**	845.10
**Task switching (switch cost)**								
Linear^a^	**304.29**	**–**	**–**	− **452.22**	**–**	**–**	914.45	**933.40**
Quadratic	302.82	1.56	0.213	− 451.43	1.58	0.209	914.87	937.61
Cubic	299.85	3.19	0.075	− 449.82	3.23	0.0722	**913.64**	940.17
**Task switching (mixing cost)**								
Linear^a^	**295.83**	**–**	**–**	− **447.62**	**–**	**–**	**905.23**	**924.18**
Quadratic	294.43	1.53	0.216	− 446.84	1.55	0.213	905.68	928.42
Cubic	293.45	1.07	0.301	− 446.30	1.09	0.296	906.59	933.12
**Tower of Hanoi (score)**								
Linear	308.21	–		− 467.49	–	–	944.97	964.15
Quadratic	289.21	22.14	< 0.001	− 456.61	21.76	< 0.001	925.21	948.22
Cubic^a^	**273.74**	**18.99**	**< 0.001**	− **447.20**	**18.80**	**< 0.001**	**908.41**	**935.25**

Bold values indicate best fitting model according to goodness-of-fit index; RSS = residual sum of squares; *F*Δ denotes the comparison of models (i.e., linear vs. quadratic); − 2LL = log-likelihood.

^a^Overall best-fitting model taking all goodness-of-fit indices into consideration.

The best fitting model for the log-transformed congruency effect in the Stroop task included linear and quadratic age coefficients. The results of the model indicated that there was a significant association between the Stroop congruency effect and age, IQ, and SES (R^2^ = 0.14, *F*(4, 335) = 13.46, *p* < 0.001). Age was significantly associated with the Stroop congruency effect (linear *β* = − 0.27, *p* < 0.001; quadratic *β* = − 0.28, *p* < 0.001). To interpret the curvilinear relationship between the Stroop congruency effect and age, we consider the model predictions displayed in Fig. 2A. Figure 2A indicates that there is some increase in the Stroop congruency effect between 10 and 35 years of age (i.e., an improvement in inhibitory control) and a decrease in the Stroop congruency effect from 36 to 86 years of age (i.e., a decline in inhibitory control). IQ was also significantly associated with the Stroop congruency effect (*β* = 0.20, *p* < 0.001), but SES was not (*β* = 0.09, *p* = 0.106). The model remained significant when IQ and SES covariates were removed (quadratic R^2^ = 0.09, *F*(2, 344) = 17.27, *p* < 0.001), showing that age was significantly associated with the Stroop congruency effect in our sample (linear *β* = − 0.19, *p* < 0.001; quadratic *β* = − 0.27, *p* < 0.001).

The best fitting model for the OSpan partial score included linear and quadratic age coefficients. The results of the model indicated that there was a significant association between the OSpan partial score and age, IQ, and SES (R^2^ = 0.28, *F*(4, 322) = 32.59, *p* < 0.001). Age was significantly associated with the OSpan partial score (linear *β* = − 0.48, *p* < 0.001; quadratic *β* = − 0.30, *p* < 0.001). To interpret the curvilinear relationship between the OSpan partial score and age, we consider the model predictions displayed in Fig. 2B. Figure 2B indicates that there is some increase in the OSpan scores from 10 to 30 years of age (i.e., an improvement in working memory capacity), and a decrease from 30 onwards (i.e., a decline in working memory capacity). IQ was also significantly associated with the OSpan partial score (*β* = 0.44, *p* < 0.001), but SES was not (*β* = 0.004, *p* = 0.930). The model remained significant when IQ and SES covariates were removed (quadratic R^2^ = 0.11, *F*(2, 331) = 20.78, *p* < 0.001), showing that age was significantly associated with the OSpan partial score in our sample (linear *β* = − 0.27, *p* < 0.001; quadratic *β* = − 0.22, *p* < 0.001).

The best fitting model for the Task Switching switch cost included the linear age coefficient. The results of the model indicated that there was a significant association between the Task Switching switch cost and age, IQ, and SES (R^2^ = 0.05, *F*(3, 323) = 5.19, *p* = 0.002). Age was significantly associated with the log-transformed switch cost (linear *β* = 0.20, *p* < 0.001), indicating a decrease in switch cost from 10 to 86 years old (i.e., an improvement in cognitive flexibility in terms of ‘switch cost’; Fig. 2D). IQ and SES were not significantly associated with switch cost (both *p*s > 0.134). The model remained significant when IQ and SES covariates were removed (linear R^2^ = 0.04, *F*(1, 331) = 14.81, *p* < 0.001), showing that age was significantly associated with the Task Switching switch cost in our sample (linear *β* = 0.21, *p* < 0.001).

The best fitting model for the Task Switching mixing cost included the linear age coefficient. The results of the model indicated that there was a significant association between the Task Switching mixing cost and age, IQ, and SES (R^2^ = 0.07, *F*(3, 323) = 8.24, *p* < 0.001). Age was significantly associated with the log-transformed switch cost (linear *β* = − 0.26, *p* < 0.001), indicating an increase in mixing cost from 10 to 86 years old (i.e., a decline in cognitive flexibility in terms of ‘mixing cost’; Fig. 2E). IQ and SES were not significantly associated with switch cost (both *p*s > 0.103). The model remained significant when IQ and SES covariates were removed (linear R^2^ = 0.07, *F*(1, 331) = 23.86, *p* < 0.001), showing that age was significantly associated with the Task Switching mixing cost in our sample (linear (*β* = − 0.26, *p* < 0.001).

The best fitting model for the Tower of Hanoi absolute score included linear, quadratic, and cubic age coefficients. The results of the model indicated that there was a significant association between the Tower of Hanoi absolute score and age, IQ, and SES (R^2^ = 0.20, *F*(5, 336) = 17.00, *p* < 0.001). Age was significantly associated with Tower of Hanoi absolute score (linear *β* = − 0.15, *p* = 0.004; quadratic *β* = − 0.25, *p* < 0.001; cubic *β* = 0.21, *p* < 0.001). To interpret the curvilinear relationship between the Tower of Hanoi absolute score and age, we consider the model predictions displayed in Fig. 2C. Figure 2C indicates that there is an initial increase in Tower of Hanoi absolute scores from 10 to 30 years of age (i.e., an increase in planning ability), a decrease from 30 to 70 years of age (i.e., a decrease in planning ability), and a small, but variable, increase from 70 years of age onwards. IQ was also significantly associated with the Tower of Hanoi absolute score (*β* = 0.43, *p* < 0.001), but SES was not (*β* = 0.005, *p* = 0.921). The model remained significant when IQ and SES covariates were removed (cubic R^2^ = 0.05, *F*(3, 344) = 6.65, *p* < 0.001), showing that age was significantly associated with the Tower of Hanoi absolute score in our sample (linear *β* = − 0.41, *p* = 0.002; quadratic *β* = − 0.25, *p* < 0.001; cubic *β* = 0.26, *p* < 0.001).

### Relationships between measures of executive functions

A series of Pearson’s correlations were conducted between the four EF tasks to investigate the relationship between the measures of EF (Table 5). Partial correlations were also conducted to control for the effects of age. These effects of age for each EF measure were determined from the previously described regression models, i.e., the EF measures were adjusted for the linear, quadratic or cubic age effects. To adjust for age, the residuals were obtained from the regression line fit when fitting each EF measure as a dependent variable in a linear model and age coefficients (linear, quadratic, or cubic age coefficients) as predictor variables.

**Table 5:** Correlation matrix of the executive function measures of interest (partial correlation coefficients controlling for the effects of age are presented in parentheses).

Measure	1	2	3	4	5
1. Stroop congruency effect^a^ (*inhibitory control*)	–				
2. Operation span partial score (*working memory*)	0.18** (0.09)	–			
3. Task switching switch cost^a^ (*cognitive flexibility*)	0.06 (0.08)	− 0.06 (− 0.02)	–		
4. Task switching mixing cost^a^ (*cognitive flexibility*)	0.02 (− 0.01)	0.12 (0.07)	− 0.46*** (− 0.43***)	–	
5. Tower of Hanoi Score (*planning*)	0.09 (0.07)	0.30*** (0.29***)	0.02 (0.03)	0.00 (0.01)	–

*p < .05, **p < .01, ***p < .001.

^a^Response time measures log-transformed and reverse scored.

The OSpan partial score showed a positive correlation with both the Stroop congruency effect and the Tower of Hanoi score, with only a relationship with the Tower of Hanoi score remaining once accounting for the effects of age. These findings suggest that individuals with a higher working memory capacity also possess better planning ability, and these relationships are present irrespective of any age effects. Finally, Task Switching switch and mixing costs showed a negative correlation, reflecting that individuals with a greater switch cost also had a smaller mixing cost, and vice versa, and this pattern remained when accounting for the effect of age.

### Comparing developmental trajectories of executive function

To examine whether each component of EF followed comparable or distinct developmental trajectories, we conducted across model comparisons for the age-related effects in the different EFs. This statistical method allows us to compare EF regression models with the same number of predictor variables, allowing direct comparisons between trajectories across these tasks. In our data, Task Switching switch and mixing cost models have three predictors (i.e., linear age coefficient, plus IQ and SES), Stroop congruency effect and OSpan partial score models have four predictors (i.e., linear and quadratic age coefficients, plus IQ and SES), and the Tower of Hanoi absolute score model has five predictors (i.e., linear, quadratic, and cubic age coefficients, plus IQ and SES). Therefore, Task Switching switch cost and mixing cost and Tower of Hanoi absolute score revealed different age-related effects (i.e., linear-only age effects vs. cubic age effect) and so were not directly compared with any other component of EF; analysis focused on the Stroop congruency effect versus OSpan partial scores.

Stroop congruency effect and OSpan partial score revealed similar curvature in the previous regression models (i.e., a quadratic effect of age), and so were directly compared. Two regression models were conducted and compared to statistically assess whether the age-related effects in the Stroop task and OSpan were significantly different. In the first step, a model was conducted that specified the outcome variable as the z-scores for the Stroop congruency effect and the OSpan partial score, with the predictor variable as the linear and quadratic age coefficients. In the second step, the same model was specified with the addition of an interaction term that included a grouping variable (i.e., a dummy variable) for the Stroop congruency effect (coded as 1) and the OSpan partial score (coded as 2). In the final step, these two models were compared using an ANOVA. If the *p* value was less than 0.05, then the regression slopes for the relationship between Stroop congruency effect and age versus OSpan partial score and age could be considered significantly different. If the *p* value was more than 0.05, then the regression slopes could be considered not statistically different.

The results indicated that the regression slopes for the Stroop congruency effect and OSpan partial score were not significantly different (RSSΔ = 2.96, *F*Δ = 1.11, *p* = 0.344), suggesting that inhibitory control and working memory show similar developmental trajectories. As illustrated in Fig. 2, the regression slopes for the other components of EF follow different patterns over age, indicating that only inhibitory control and working memory have similar developmental trajectories and all other components of EF show distinct developmental trajectories.

## Discussion

The current study explored age-related differences in EF from late childhood through to old age in a large, community-based sample. Three-hundred and fifty individuals aged 10 to 86-years-old completed tasks to measure inhibitory control, working memory, cognitive flexibility, and planning, to identify when age-related changes in these EFs first become apparent. After controlling for any potential effects of IQ and SES, analyses revealed that inhibitory control and working memory capacity was higher in young adulthood compared to adolescence, with inhibitory control showing a decline in participants from ~ 35-years-old, and working memory capacity showing a decline in participants from ~ 30-years-old. Planning ability was also higher in young adulthood compared to adolescence, but then declined across adulthood, with a small positive change in older age. In line with our hypothesis, a dissociation was found for the measures of cognitive flexibility: interestingly, however, this reflected that switch costs decreased across the lifespan, yet mixing costs increased across the lifespan.

These findings provide insight into the developmental trajectories of inhibitory control, working memory, cognitive flexibility, and planning ability across the lifespan, providing a more comprehensive picture of the age-related changes in EF than has previously been established. Many of the existing studies that have examined aging and EFs have compared a dichotomous sample of younger versus older adults (e.g.,^51,93–95^), have combined individuals into smaller age groups during analysis (e.g.,^53,55^), or have focused on single aspects of EF, such as inhibitory control (e.g.,^19,23^). Instead, in the current study, we used a continuous age sample to model curvilinear age relationships to show the development of EFs from adolescence through to older adulthood, and to highlight changes in EFs that emerge throughout adulthood and not specifically at the onset of old age (typically considered 65 years old plus). Studies have largely overlooked adulthood as a period of change, with many studies omitting middle-aged adults in their samples examining lifespan changes. Moreover, cognitive performance among adolescents has rarely been compared to middle- or older-aged adults. The current study therefore makes a unique contribution to the literature by demonstrating developmental changes in different EFs, using the same set of tasks for all participants, with evidence that declines emerge in inhibitory control, working memory, and planning as early as the third decade of life. In addition, inhibitory control and working memory follow comparable developmental trajectories, with distinct developmental trajectories apparent for the other measures of EF.

In line with our predictions, and supporting previous studies^61^, the current study highlighted that different aspects of cognitive flexibility showed distinct age effects. As expected, there was an increase in mixing costs across adulthood, but switch costs *decreased* across adulthood. Mixing costs have generally been found to be greater in older adults (e.g.,^54–58^) there are mixed results in regard to switch costs, with some studies reporting an age-related increase (e.g.,^59^), a U-shaped trajectory^53^ or no age-related differences (e.g.,^58,59^), most likely due to differences in the task switching paradigms. We note that the current study used an alternating-runs paradigm without a preparatory cue-stimulus interval, which is analogous to Huff et al.’s^84^ task-switching paradigm with comparable aging results. In addition, switch and mixing costs showed a negative correlation, reflecting that individuals with a greater switch cost also had a reduced mixing cost, and vice versa, and this pattern also remained when accounting for the effect of age. This finding replicates that seen in Huff et al.^84^ in which a dissociation was found between switch and mixing costs across age groups. Huff et al.^84^ suggested that this dissociation is due to differences in the attentional systems in younger versus older adults. They suggest that younger and middle-aged adults experience a larger switch cost as their attentional systems become tuned to the task set in the single-task, and this inertia to executing the same rule in the single-task slows the reconfiguration to respond to the switch trials in the mixed-task. Older adults experience a reduced switch cost as their attentional systems are less well tuned to the task set in the single-task, and so do not experience the same slow down to respond to switch trials in the mixed-task. Moreover, older adults experience a larger mixing cost due to the additional attentional demands of maintaining two task sets in the mixed-task as compared to a single task set in the single-task. In summary, results of the task switching paradigm demonstrate dissociations between switch and mixing costs across the lifespan, indicating that adolescents and younger adults have more difficulty switching between task sets, and middle-aged and older adults have more difficulty maintaining task sets.

In the current study, we utilized four widely used tasks to measure inhibitory control, working memory, cognitive flexibility, and planning as components of EF. We investigated the relationship between these measures and found that individuals with a higher working memory capacity also had better planning ability, and these relationships remained when accounting for the effects of age. This finding suggests a link between working memory capacity and planning ability, or alternatively it could suggest that some EF tasks purported to measure singular aspects of EF may also require other EF processes to complete. This is supported by prior literature which has suggested that ‘planning’ may be indicative of a more complex executive skill, requiring activation of other aspects of EF to be successful (e.g.,^26,96,97^). For instance, working memory may be required when utilizing planning abilities to allow thinking ahead and execution of steps to achieve a set goal^26,97^; Hill and Bird^98^ also suggest that the traditional tower tasks (as used here to assess planning abilities) may require working memory, the inhibition of prepotent responses, and the generation of problem-solving ideas.

Interestingly, there were no other relationships between the measures of EF. Other research has documented very weak relationships between EF tests and EF factors, leading to the conclusion that these are dissociable components of EF and providing support for the fractionated EF theory (e.g.,^83,97,99^). Studies that do report relationships between components of EF tend to use several different EF tasks to assess each component and use an SEM approach to fit and compare models. For instance, Miyake et al.^83^ report in their study that, following completion of nine tasks used to assess shifting, updating, and inhibition, a three-factor model fitted the data best, highlighting distinguishable factors of: cognitive flexibility (shifting), updating, and inhibition. In the current study, we did not aim to assess whether EF is a unitary or diverging construct, and as such data is not optimised to investigate this specifically. However, the lack of correlations between tasks in the current data suggest that the tasks are tapping into distinguishable capacities rather than ‘umbrella’ EFs. Furthermore, EFs differentiate from middle to late childhood^100^. Our study is unique in exploring four separate measures of EFs (as opposed to an aggregated measure of cognitive performance), allowing across model comparisons which revealed that inhibitory control and working memory follow similar developmental trajectories, and all other measures of EF show distinct developmental trajectories.

In general, there is no single task or task battery that can exhaustively measure all aspects of EF, and tests of individual EF are rarely “process pure”^97,101^. Furthermore, there is some debate about whether tasks measure the underlying concept that they are purported to measure. For example, it has been suggested that participants may solve the Tower of Hanoi problems in a step-by-step manner instead of in a multi-step, planful manner^102^. It is also likely that the specific processes involved in each task differ across individuals and cohorts. For example, the method of administration used in the OSpan task here (i.e. requiring participants to select their answer from letters in a 4 × 3 grid) is likely to have differentially affected performance among the older participants since they are less familiar with computers and are known to experience age-related difficulties in visual search tasks and motor control (e.g.,^103,104^). In addition, it is noted that we used a single task to measure each component of EF. There may be specific aspects of each EF that may follow different developmental trajectories—for example, inhibitory control could be divided into automatic and effortful inhibition^105^. However, the aim of the current study was to examine how four separable EFs (inhibitory control, working memory, cognitive flexibility, and planning) may continue to change and differ across the lifespan, to further our understanding of age-related cognitive changes that may be present; to do this, we selected four well-established tasks that were suitable for use across the participant sample age-range, 10–86 years. This allowed direct comparison of task performance across different participant ages. It is noted, as previously recommended^106^, that in future studies it would be beneficial to include multiple measures of each component of EF to elucidate whether these age-related changes reflect the underlying EF or whether the age-related effects are task- or paradigm-specific. Furthermore, dual-tasks of EF may reveal greater age-related declines as multiple EFs are loaded in a single task; for example, loading working memory in younger adults has been found to reduce both inhibitory control and switching ability^107^. Tasks need to be sensitive enough to detect age-related declines^108^ and should account for general cognitive processing^61^. The four EF tasks in the current study were found to be age-sensitive after adjusting for general cognitive declines in response latencies and for IQ and SES, and therefore suitably provide an overall lifespan description of EF. We note that our analyses did not factor in the influence of gender on EFs, though gender was unequally distributed across the age groups in our sample (e.g., 47% females among adolescents but 82% females among adults). Previous research has provided mixed evidence for gender or sex differences in executive functioning (e.g.,^109^), and these analyses were beyond the scope of the current paper, however it would be beneficial for future studies to systematically explore this influence further. Gender details in our sample are available alongside task data on the OSF repository.

Here, we describe the overall developmental trajectories of EFs. To increase confidence in findings relating to this main aim, we controlled for any effects of IQ and SES when exploring age effects on EFs, due to evidence suggesting that some components of EF may be related to IQ and SES^54,56,67^. For IQ measures, our results highlighted a relationship with inhibitory control, working memory, and planning ability, above the effects of age. This may also explain why, in our measure of planning ability, a small, variable, improvement in abilities is seen from 70 years old onwards. Notably, the older age participants who took part in this study had higher IQ scores (full scale, verbal, and performance) than any other age group included in analysis; participants in this study were community-based, and this higher IQ may reflect that those experiencing the optimum ‘healthy’ aging experience are more likely to agree to take part in research studies such as these. This provides insight into healthy aging processes and may indicate that IQ holds a protective role against age-related declines in EF, although further research aimed at directly examining this suggestion, particularly its role in predicting planning abilities, is required. It is interesting to note that in this study, SES was not related to any component of EF, above the effects of IQ and age. So far, no other study has examined current SES on adult EF; literature has instead used a longitudinal approach to examine how SES during a distinct period (typically childhood) predicts later EF (e.g.,^110^). The current findings therefore suggest that an individual’s SES can change over the lifespan, which may have an additional effect on cognition^111^, and that SES may be less critical for EF after childhood.

It is interesting to note that not every individual demonstrated the same developmental profile of EF; for example, some older adults show equivalent performance in tasks to younger adults. The current study used a cross-sectional design to identify when age-related differences emerge when examining performance on four key measures of EF abilities. Given the scope of this design, results can only assess group-level age-related changes. Cross-sectional studies are potentially confounded by cohort effects and might therefore overestimate age-related changes, potentially failing to accurately explore age-related changes in task performance at an individual-level (i.e., how an individual’s EF capacities change over time; see^112^). For instance, prior studies using longitudinal analysis have highlighted that during middle age (i.e., 20–60 years), cognitive abilities such as speed of processing decline, but at a smaller rate than may be indicated in cross-sectional analyses (e.g.,^113^). The current study provides insight into the presence of age-related differences in EF abilities across the lifespan using a cross-sectional approach; it would be of interest in future to further this research by utilizing longitudinal designs to furthering our understanding of how EFs change with age, and individual differences that may influence these changes. It is also noted that the current sample consisted of a community sample of healthy adult volunteers functioning at high levels and may therefore, as discussed above, represent ‘successful’ aging within this particular population. There may be other factors that influence an individual’s performance on the EF tasks over and above age-related effects, which would be of interest to examine in future research; for example, there may be protective factors that offset declines in EFs, such as increased cardiovascular fitness in older age relating to better inhibitory control^114^.

As previously stated, EFs play an important role in daily life. Poor EFs can lead to social problems^80,81^, obesity and overeating^79,115^, lower productivity and difficulty keeping a job^82^, and people with better EF abilities have been shown to enjoy an improved quality of life^78^. Diamond^1^ highlights the importance of EFs for maintenance of mental and physical health. Given this, it is important to further our understanding of how EF abilities continue to change and evolve across the lifespan, examining not only childhood/adolescence and older adulthood, but observing differences across all of adulthood. Furthering prior research that has sought to establish changes in EFs across the lifespan (e.g.,^40,42,48^; see also^41^), the current study used four tasks to assess key EF abilities, including inhibitory control, working memory, cognitive flexibility, and planning abilities, providing further insight into cross-sectional changes seen in EF abilities across the lifespan. EF is a ‘functional construct’, involved in helping individuals conduct deliberate, goal-directed thoughts and actions^48^; by examining which aspects of EF do or do not change across the lifespan, and which tasks are able to sensitively assess differences in EF abilities across different ages, we are able to gain information about the overall EF construct. The tasks used in the current study were shown to be suitable for use with individuals from ten to 86 years of age, sensitively detecting differences in EF abilities. Additionally, by identifying the ages at which changes in EFs are seen, we may be able to develop targeted interventions to help maintain efficient EF capacities, in turn assisting in increased success in real-world scenarios. By analysing the data in the current study as a continuous sample, allowing curvilinear relationships to be examined, results highlight changes in EF abilities can be observed from young adulthood, and emphasise the importance of looking at all ages when examining cognitive changes, rather than focussing on ‘younger’ versus ‘older’ age groups.

## Conclusion

We explored developmental changes in inhibitory control, working memory, cognitive flexibility, and planning ability from 10 years old to 86 years old in a large, community-based sample of healthy individuals. We show that working memory capacity and planning ability continue to develop over adolescence and into early adulthood. Crucially, we show that declines emerge as early as the third decade of life in inhibitory control, working memory, and planning, which is much earlier than has previously been considered. In addition, we demonstrate a dissociation for measures of cognitive flexibility, with switch costs decreasing and mixing costs increasing up to older age, indicating that adolescents and young adults have difficulties switching tasks sets, whereas middle-aged and older adults have difficulties maintaining task sets. In general, studies have largely overlooked adulthood as a period of change in EFs, with studies focussing on their development in childhood, or comparing dichotomous groups of young versus older adults in studies of cognitive aging. The findings of the current study highlight the value of including adolescents and middle-aged adults to provide a comprehensive lifespan description of the distinct developmental trajectories of EFs.

## Supplementary information

Supplementary Information.
