# Supplementary material for: The developmental trajectories of executive function from adolescence to old age
Source: Sci Rep. 2021 Jan 14;11:1382. doi: 10.1038/s41598-020-80866-1 (PMC7809200; doi:10.1038/s41598-020-80866-1)
Supplement: Supplementary file 1 — Supplementary Information. [file 41598_2020_80866_MOESM1_ESM.docx]

**Journal**: Scientific Reports

**The developmental trajectories of executive function from adolescence to old age**

Victoria E. A. Brunsdon^1^, Elisabeth E. F. Bradford^2^, & Heather J. Ferguson^1^*†

^1^ School of Psychology, University of Kent, UK

^2^ School of Psychology, University of Dundee, UK

7,991 words (excluding references, tables and figures)

*Author for correspondence (h.ferguson@kent.ac.uk).

†Present address: School of Psychology, University of Kent, Canterbury, CT2 7NP, UK.

Acknowledgements

This work was carried out with the support of a European Research Council grant to HF (Ref: CogSoCoAGE; 636458). The datasets and code supporting this article are available on the Open Science Framework (https://osf.io/qzrwu). VB contributed to study design, data collection, data analysis and interpretation, and drafting the manuscript; EB contributed to study design, data collection, and revising the manuscript; HF conceived of the study, designed the study, and revised the manuscript. All authors gave final approval for publication.

# Supplementary Materials 1

| Supplementary Table 1.  Goodness-of-fit indices for the executive function models (untransformed variables) with age as the predictor variable (with linear, quadratic, or cubic age coefficients) for each outcome variable. | | | | | | | | | |
| --- | --- | --- | --- | --- | --- | --- | --- | --- | --- |
| Model | | Model Fit Indices | | | | | | | |
|  | | ANOVA | | | Likelihood Test | | | AIC | BIC |
|  | | RSS | F | p | -2LL | 𝜒2 | p |  |  |
| Stroop Task (Congruency Effect) | |  |  |  |  |  |  |  |  |
|  | Linear | 303.30 | — | — | -463.02 | — | — | 936.05 | 955.19 |
|  | Quadratic* | **259.75** | **56.17** | **< .001** | **-436.67** | **52.71** | **< .001** | **885.34** | **908.31** |
|  | Cubic | 259.75 | 0.00 | .967 | -436.67 | 0.00 | .966 | 887.34 | 914.14 |
| Task Switching (Switch Cost) | |  |  |  |  |  |  |  |  |
|  | Linear | 324.31 | — | — | -462.64 | — | — | 935.28 | 954.23 |
|  | Quadratic* | **314.41** | **10.14** | **.002** | **-457.57** | **10.13** | **.001** | **927.15** | 949.89 |
|  | Cubic | 314.41 | 0.00 | .957 | -457.57 | 0.00 | .956 | 929.14 | 955.67 |
| Task Switching (Mixing Cost) | |  |  |  |  |  |  |  |  |
|  | Linear* | **295.95** | **—** | **—** | **-436.24** | **—** | **—** | 882.48 | **901.43** |
|  | Quadratic | 275.95 | 0.00 | .981 | -436.24 | 0.00 | .980 | 884.48 | 907.22 |
|  | Cubic | 272.17 | 4.46 | .035 | -433.98 | 4.51 | .034 | **881.96** | 908.49 |
| Note: bold values indicate best fitting model according to goodness-of-fit index; RSS = residual sum of squares; *F*Δ denotes the comparison of models (i.e., linear vs. quadratic); -2LL = log-likelihood; * = overall best-fitting model taking all goodness-of-fit indices into consideration | | | | | | | | | |

# Supplementary Materials 2

**Untransformed DV Models**

The best fitting model for the untransformed congruency effect in the Stroop task included linear and quadratic age coefficients. The results of the model indicated that there was a significant association between the Stroop congruency effect and age, IQ, and SES (R^2^ = .25, *F*(4, 335) = 27.16, *p* < .001). Age was significantly associated with the Stroop congruency effect (linear *β* = -.27, *p* < .001; quadratic *β* = -.28, *p* < .001). To interpret the curvilinear relationship between the Stroop congruency effect and age, we consider the model predictions displayed in Supplementary Figure 1A. Supplementary Figure 1A indicates that there is some increase in the Stroop congruency effect between 10 years and 40 years of age (i.e., an improvement in inhibitory control) and a decrease in the Stroop congruency effect from 40 to 86 years of age (i.e., a decline in inhibitory control). IQ was also significantly associated with the Stroop congruency effect (*β* = .20, *p* < .001), but SES was not (*β* = .09, *p* = .329).

The best fitting model for the untransformed Task Switching switch cost included linear and quadratic age coefficients. The results of the model indicated that there was a significant association between the Task Switching switch cost and age, IQ, and SES (R^2^ = .04, *F*(4, 322) = 2.99, *p* = .019). Age was significantly associated with the Task Switching switch cost (linear *β* = -.05, *p* = .394; quadratic *β* = -.18, *p* = .002). To interpret the curvilinear relationship between the Task Switching switch cost and age, we consider the model predictions displayed in Supplementary Figure 1B. Supplementary Figure 1B indicates that there is some increase in the Task Switching switch cost between 10 years and 50 years of age (i.e., an improvement in cognitive flexibility in terms of ‘switch cost’) and a decrease in the Task Switching switch cost from 50 to 86 years of age (i.e., a decline in cognitive flexibility in terms of ‘switch cost’). IQ and SES were not significantly associated with the Task Switching switch cost (both *p*s > .329).

The best fitting model for the untransformed Task Switching mixing cost included linear-only age coefficients. The results of the model indicated that there was a significant association between the Task Switching mixing cost and age, IQ, and SES (R^2^ = .13, *F*(3, 323) = 16.79, *p* < .001). Age was significantly associated with the Task Switching mixing cost (linear *β* = -.38, *p* < .001). To interpret the curvilinear relationship between the Task Switching mixing cost and age, we consider the model predictions displayed in Supplementary Figure 1C. Supplementary Figure 1C indicates that there is a decrease in Task Switching mixing cost between 10 years and 86 years of age (i.e., i.e., a decline in cognitive flexibility in terms of ‘mixing cost’). IQ and SES were not significantly associated with the Task Switching mixing cost (both *p*s > .233).

| 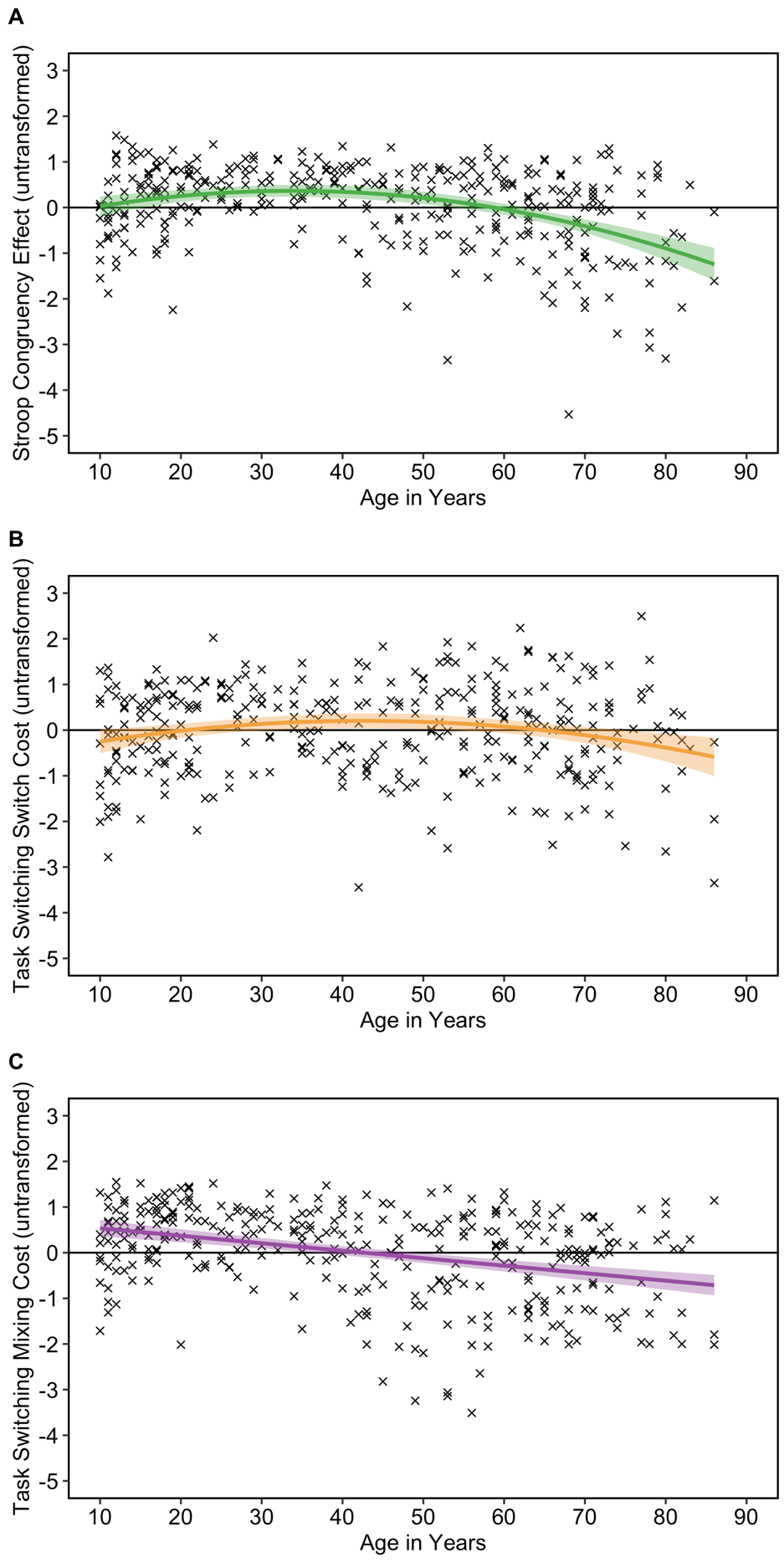 |
| --- |
| Supplementary Figure 1.  Relationship between age and executive function measures. (A) untransformed Stroop congruency effect, (B) untransformed Task Switching switch cost; and (C) untransformed Task Switching mixing cost. The bold line indicates the best-fitting regression line and the dashed line indicates the 95% confidence intervals (CIs). All variables are reversed scored so that a higher value indicates better performance. All variables are z-scored for ease of comparison. |
